# Supplementary material for: Evaluation of the Hamburg-Glasgow Classification in Pancreatic Cancer: Preoperative Staging by Combining Disseminated Tumor Load and Systemic Inflammation
Source: Cancers (Basel). 2021 Nov 25;13(23):5942. doi: 10.3390/cancers13235942 (PMC8657182; doi:10.3390/cancers13235942)
Supplement: Supplementary file 1 [file cancers-13-05942-s001.zip › cancers-1465605-supplementary.pdf]

Supplementary Materials: Evaluation of the Hamburg-Glasgow Classification in Pancreatic Cancer: Preoperative Staging by Combining Disseminated Tumor Load and Systemic Inflammation

Thaer S. A. Abdalla, Valeria Almanfalouti, Katharina Effenberger, Faik G. Uzunoglu, Tarik Ghadban, Anna Dupreé, Jakob R. Izbicki, Klaus Pantel and Matthias Reeh

1. Supplementary Figures

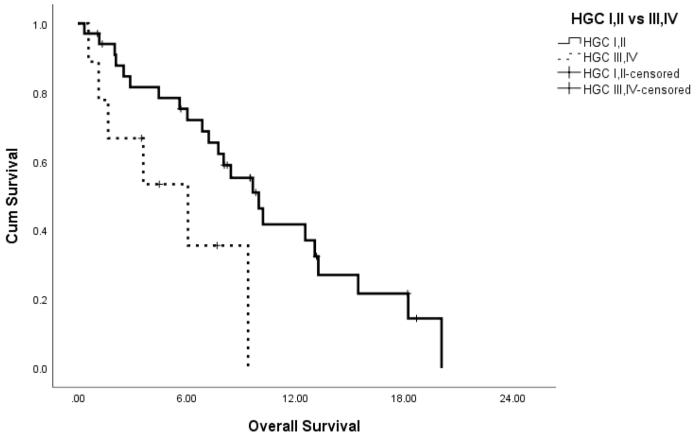

Overall comparison	Chi-Square	p
Log Rank (Mantel-Cox)	4.720	0.030

Legend: p-value Indicates significance according to Log-Rank (Mantel-Cox).

Figure S1. Univariate Kaplan-Meier analysis for overall survival according to HGC in metastatic pancreatic cancer. Legend: p-value Indicates significance according to Log-Rank (Mantel-Cox).

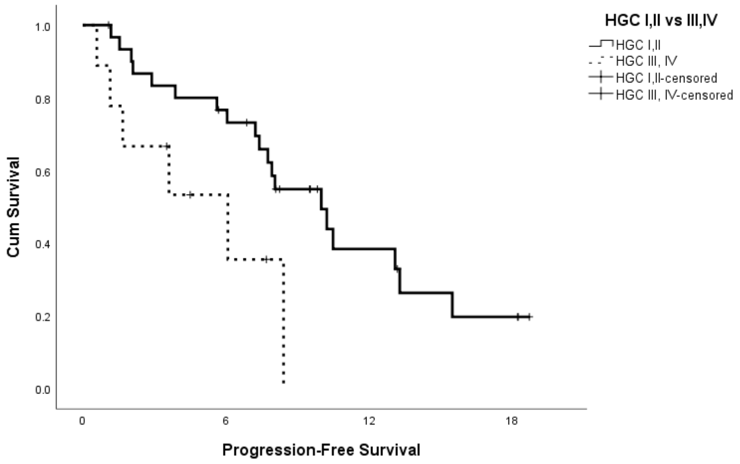

Overall comparison	Chi-Square	p
Log Rank (Mantel-Cox)	5.051	0.025

Legend: p-value Indicates significance according to Log-Rank (Mantel-Cox).

Figure S2. Univariate Kaplan-Meier analysis for progression-free survival according to HGC in metastatic pancreatic cancer.

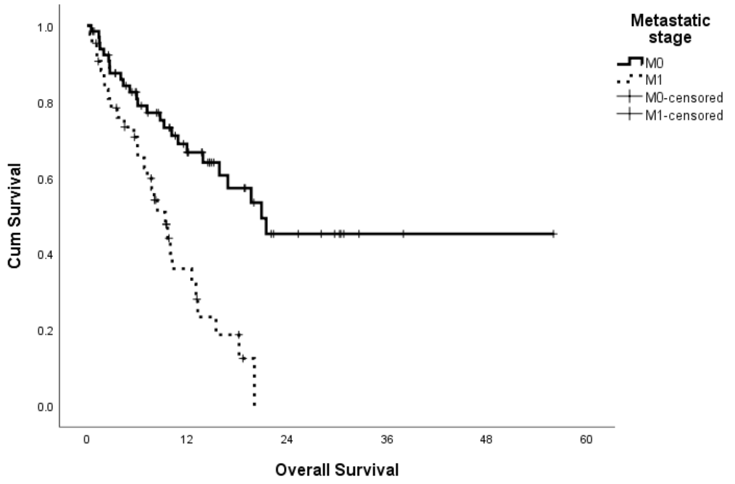

Overall comparison	Chi-Square	*p*
Log Rank (Mantel-Cox)	15.735	≤0.001

Legend: *p*-value Indicates significance according to Log-Rank (Mantel-Cox).

Figure S3. Univariate Kaplan-Meier analysis for overall survival according to the metastatic status.

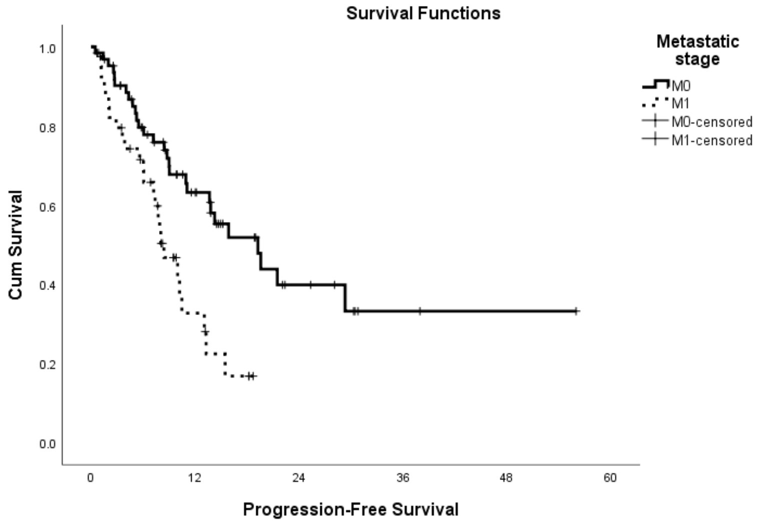

Overall comparison	Chi-Square	*p*
Log Rank (Mantel-Cox)	9.579	0.002

Legend: *p*-value Indicates significance according to Log-Rank (Mantel-Cox).

Figure S4. Univariate Kaplan-Meier analysis for progression-free survival according to the metastatic status.

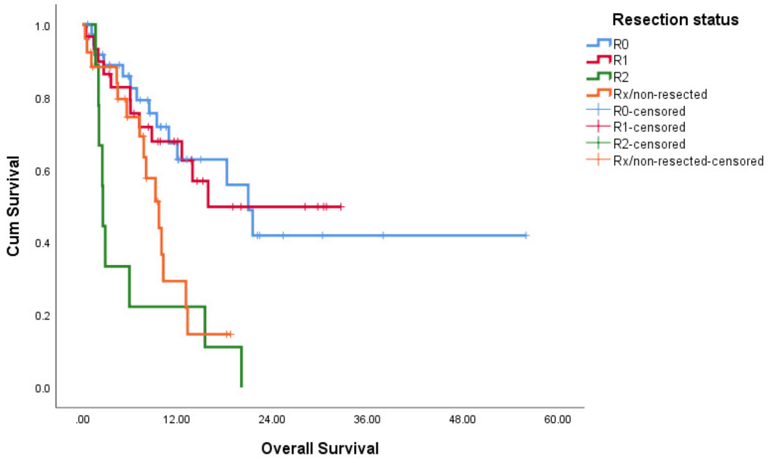

Overall comparison	Chi-Square	*p*
Log Rank (Mantel-Cox)	20.506	<0.001

Pair-wise comparison	*p*
R2 vs R1	0.001
R2 vs R0	<0.001
R2 vs Rx	0.190

Legend: *p*-value Indicates significance according to Log-Rank (Mantel-Cox).

Figure S5. Univariate Kaplan-Meier analysis for overall survival according to Resection status.

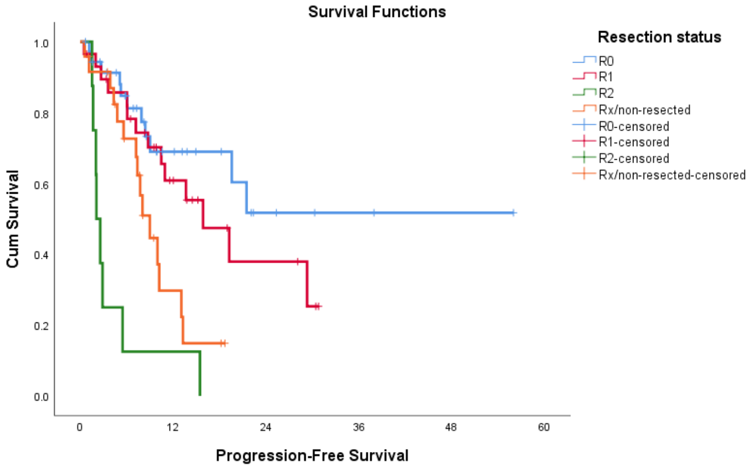

Overall comparison	Chi-Square	*p*
Log Rank (Mantel-Cox)	32.320	<0.001

Pair-wise comparison	*p*
R2 vs R1	<0.001
R2 vs R0	<0.001
R2 vs Rx	0.03

Legend: *p*-value Indicates significance according to Log-Rank (Mantel-Cox).

Figure S6. Univariate Kaplan-Meier analysis for progression-free survival according to Resection.

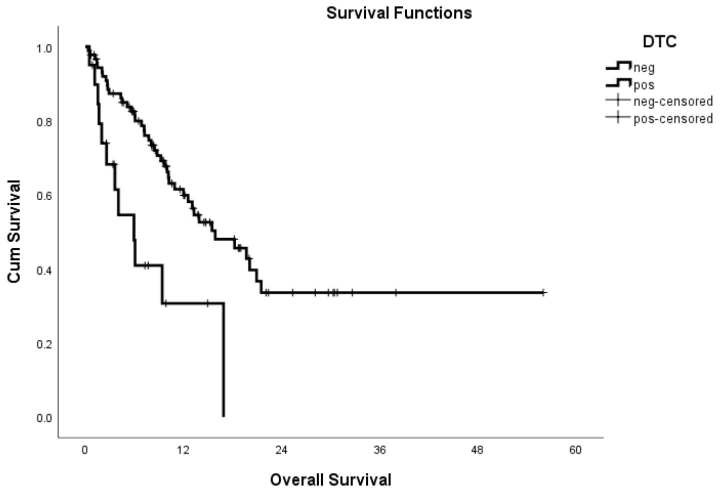

Overall comparison	Chi-Square	*p*
Log Rank (Mantel-Cox)	10.585	0.001

Legend: *p*-value Indicates significance according to Log-Rank (Mantel-Cox).

Figure S7. Univariate Kaplan-Meier analysis for overall survival according to DTC detection.

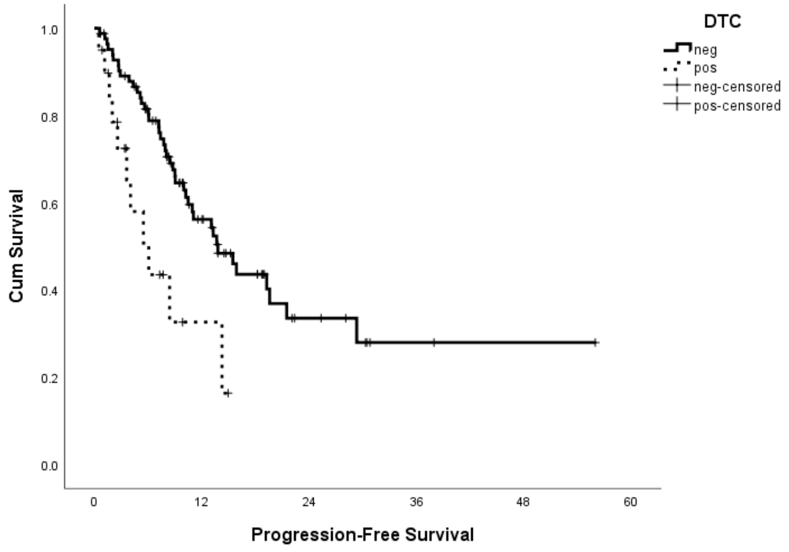

Overall comparison	Chi-Square	*p*
Log Rank (Mantel-Cox)	8.267	0.004

Legend: *p*-value Indicates significance according to Log-Rank (Mantel-Cox).

Figure S8. Univariate Kaplan-Meier analysis for progression-free survival according to DTC detection.

2. Supplementary Tables

Table S1. Multivariate analysis of overall survival in patients with pancreatic cancer without stratification.

Variables	Multivariate Analysis		
	HR	95% Cl	*p*

Age,<br><65, ≥65	1.96	1.09–3.52	0.024
Sex,<br>male vs female	1.40	0.78–2.50	0.252
Tumor size,<br>T1–4	1.15	0.75–1.78	0.503
Nodal status,<br>neg vs. pos	2.64	1.11–6.25	0.027
UICC,<br>I–IV	1.30	0.98–1.73	0.064
HGC,<br>I–IV	2.09	1.52–2.86	<0.001
Resection margin,<br>R0/R1 vs R2/Rx	1.51	0.79–2.88	0.212

Legend: *p* Indicates significance according to cox regression analysis comparing the specified variables. HR indicates hazard ratio.

Table S2. Multivariate analysis of progression-free survival in patients with pancreatic cancer without stratification.

Variables	Multivariate Analysis		
	**HR**	**95% Cl**	***p***
Age,<br><65, ≥65	2.21	1.20–4.09	0.011
Sex,<br>male vs female	1.34	0.75–2.40	0.320
Tumor size,<br>T1–4	1.27	0.80–2.02	0.307
Nodal status,<br>neg vs. pos	1.90	0.86–4.18	0.109
UICC,<br>I–IV	1.13	0.84–1.51	0.414
HGC,<br>I–IV	2.16	1.56–3.00	<0.001
Resection margin,<br>R0/R1 vs R2/Rx	2.01	1.03–3.92	0.039

Legend: *p* Indicates significance according to cox regression analysis comparing the specified variables. HR indicates hazard ratio.

Table S3. Multivariate analysis of overall survival in patients with pancreatic cancer when taking chemotherapy into consideration ($n = 51$).

Variables	Multivariate Analysis		
	**HR**	**95% Cl**	***p***
Age,<br>*<65, ≥65*	1.45	0.42–4.94	0.548
Sex,<br>male vs female	1.32	0.42–4.18	0.627
Tumor size,<br>T1–4	1.47	0.82–3.74	0.147
Nodal status,<br>neg vs. pos	0.315	0.06–1.54	0.154
UICC,<br>I–IV	2.73	1.48–5.06	0.001
HGC,			0.002

IV vs I	0.13	0.001–0.49	≤0.001
IV vs II	0.25	0.001–0.65	≤0.001
IV vs III	0.78	0.001–0.13	≤0.001
Resection margin, R0/R1 vs R2/Rx	7.86	1.81–34.02	0.006
Chemotherapy	0.18	0.03–1.00	0.051

Legend: *p* Indicates significance according to cox regression analysis comparing the specified variables. HR indicates hazard ratio.

Table S4. Multivariate analysis of progression-free survival in patients with pancreatic cancer when taking chemotherapy into consideration.

Variables	Multivariate Analysis		
	HR	95% Cl	*p*
Age, <65, ≥65	2.18	0.72–6.53	0.164
Sex, male vs female	1.83	0.65–5.12	0.245
Tumor size, T1–4	1.54	0.82–3.74	0.247
Nodal status, neg vs. pos	0.62	0.15–2.48	0.506
UICC, I–IV	1.52	0.93–2.48	0.093
HGC,			0.004
IV vs I	0.13	0.001–0.49	≤0.001
IV vs II	0.25	0.001–0.65	0.002
IV vs III	0.78	0.001–0.13	0.002
Resection margin, R0/R1 vs R2/Rx	7.86	1.81–34.02	0.011
Chemotherapy	0.50	0.11–2.17	0.359

Legend: *p* Indicates significance according to cox regression analysis comparing the specified variables. HR indicates hazard ratio.
